# EZH2 Is Associated with Malignant Behavior in Pancreatic IPMN via p27^Kip1^ Downregulation

**DOI:** 10.1371/journal.pone.0100904

**Published:** 2014-08-01

**Authors:** Hideyuki Kuroki, Hiromitsu Hayashi, Hirohisa Okabe, Daisuke Hashimoto, Hiroshi Takamori, Osamu Nakahara, Shigeki Nakagawa, Yukiko Fukushima, Akira Chikamoto, Toru Beppu, Masahiko Hirota, Ken-ichi Iyama, Hideo Baba

**Affiliations:** 1 Department of Gastroenterological Surgery, Graduate School of Medical Sciences, Kumamoto University, Kumamoto, Japan; 2 Department of Surgery, Kumamoto Regional Medical Center, Kumamoto, Japan; 3 Department of Surgical Pathology, Graduate School of Medical Sciences, Kumamoto University, Kumamoto, Japan; Roswell Park Cancer Institute, United States of America

## Abstract

**Background:**

The epigenetic mechanism of tumorigenesis in pancreatic intraductal papillary mucinous neoplasm (IPMN) remains largely unknown. The aim of this study is to examine the role of enhancer of zeste homologue 2 (EZH2) alteration in pancreatic IPMN progression.

**Methods:**

Fifty-four surgically resected pancreatic IPMN specimens, including a total of 181 lesions (normal duct in 48, adenoma in 50, borderline atypia in 53, carcinoma in situ (CIS) in 19, and invasive carcinoma in 11) were analyzed by immunohistochemical staining (EZH2, Ki-67, p27^Kip1^). Using paraffin embedded sections, total RNA was successfully extracted from 20 IPMN lesions (borderline IPMN in 9, CIS in 6, invasive carcinoma in 5) and 7 pancreatic normal ducts, and then levels of *EZH2* and *p27^Kip1^* mRNA were analyzed by real time PCR.

**Results:**

In immunohistochemical analysis, cell proliferative activity revealed by Ki-67 positive nuclei was increased during IPMN progression (normal duct<adenoma<borderline atypia<CIS ≈ invasive carcinoma). EZH2 expression displayed a similar pattern (normal duct<adenoma<borderline atypia<CIS ≈ invasive carcinoma) with cell proliferative activity. EZH2 expression in malignant (CIS and invasive carcinoma) IPMNs was significantly higher than that in adenoma and borderline-atypia IPMNs. EZH2 expression level in IPMN lesions was positively correlated with the Ki-67 positive nuclear ratio (*p*<0.0001). EZH2-positive cells in malignant IPMN did not express p27^Kip1^. *EZH2* mRNA expressions in malignant lesions were significantly higher than those in benign lesions (*p*<0.0001). In contrast, *p27^Kip1^* mRNA in malignant lesions was significantly decreased compared to those in benign lesion (*p*<0.05), and there was an inverse correlation between *EZH2* and *p27^Kip1^* mRNA levels (*p* = 0.0109).

**Conclusion:**

EZH2 is associated with the accelerated cell proliferation and malignant step in pancreatic IPMN via the downregulation of p27^Kip1^.

## Introduction

Cystic pancreatic lesions have been shown in up to 25% of in autopsy cases; 5% of these lesions are neoplastic, such as intraductal papillary mucious neoplasms (IPMNs) and mucinous cystic neoplasms [1], [2]. Recently, pancreatic IPMN has been clinically highlighted because of its frequent and incidental diagnosis by advanced radiological examination. Histologic examination of pancreatic IPMNs exhibits the co-existence of benign and malignant lesions within a single specimen, represented by multifocality [3], and the cases with invasive components in pancreatic IPMN exhibit a poor prognosis [4]–[7]. Thus, pancreatic IPMNs are characterized by multifocal and malignant potential represented by an adenoma-carcinoma sequence [8], [9]. However, its molecular mechanism of oncogenesis remains largely unknown due to the limited research tools, such as the availability of only resected human specimens.

Oncogenesis is a complex process associated with the accumulation of genetic and epigenetic defects that alter the transcriptional program. Pancreatic IPMNs harbor genetic alterations of KRAS [10], [11], p16^INK4^ [12], p53 [13], and DPC4/SMAD4/MADH4 [12]. However, the epigenetic mechanism of tumorigenesis in pancreatic IPMN remains largely unknown. Epigenetic gene silencing in cancer is now increasingly recognized as a novel therapeutic target [14]–[18], because uncovering its molecular events could lead to the development of clinical strategies to reverse the epigenetic gene silencing process and prevent cancer progression. Proteins of the polycomb repressive complex 2 (PRC2) function as transcriptional repressors by methylating histone H3 at lysine 27, and this complex’s activity is essential for cellular proliferation, differentiation, and cell fate decisions. The histone methyltransferase enhancer of zeste 2 (EZH2), a key member of PRC2, is associated with transcriptional repression [19]. Indeed, EZH2 acts mainly as a gene silencer in the progression of cancers such as breast [20] and prostate cancer [19], [21], and the overexpression of EZH2 is associated with aggressive and metastatic disease. In relation to pancreatic tumor, it has been reported that loss of EZH2 results in impaired pancreatic regeneration and accelerates pancreatic intraepithelial neoplasia (PanIN) in vivo [22]. Although several genetic alternations promote tumor progression in pancreatic IPMNs, the role of epigenetic alteration in pancreatic IPMN progression is unclear. Here, we report that EZH2 is associated with the accelerated cell proliferation and malignant step in pancreatic IPMN via the gene silencing of p27^Kip1^.

## Materials and Methods

From 2002 to 2011, 54 patients with pancreatic IPMN underwent curative resection in the Department of Gastroenterological Surgery, Graduate School of Medical Sciences, Kumamoto University and Department of Surgery, Kumamoto Regional Medical Center. All patients provided written informed consent for the use of their resected tissues for clinical study. This study was approved by the Human Ethics Review Committee of the Graduate School of Medicine, Kumamoto University (Kumamoto, Japan). The Clinicopathological features of the 54 patients are shown in **Table 1**. This study analyzed 181 lesions, including 133 IPMN lesions and 48 normal duct epithelial lesions, obtained from the 54 surgically resected specimens. The pathological diagnoses, such as adenoma, borderline atypia, carcinoma in site (CIS), and invasive carcinoma were reviewed and established based on the 2010 World Health Organization (WHO) classification for IPMN. Then, the 133 IPMN lesions were classified as either non-malignant IPMN (adenoma and borderline atypia) or malignant IPMN (CIS and invasive cancer).

**Table 1:** Clinicopathological features of 54 cases with IPMN.

Final diagnosis at the most advanced lesion					
	non-malignant IPMN (n = 31)	Malignant IPMN (n = 23)		Total	P-value
	Borderline atypia	CIS (n = 11)	Invasive carcinoma (n = 12)		
Gender					
Male	21	6	7	34	
Female	10	5	5	20	0.68
Age (years)	69.0±9.6	67.8±11.6	71.9±5.4	68.8±10.3	0.69
Type					
Main duct type	6	5	9	20	
Branch type	19	5	2	26	
Combined type	6	1	1	8	0.0165
Diameter of main duct (mm)	6.0±5.0	6.0±3.9	13.0±8.8	8.0±6.0	0.0547
Diameter of cyst (mm)	30.7±21.62	29.4±16.5	39.0±19.4	32.0±19.9	0.5672
Mural nodule (mm)	9.1±5.3	15.8±15.5	65.6±125.9	25.6±66.9	0.1341

Values are mean ± s.d. CIS, carcinoma in situ.

### Immunohistochemical staining

Immunohistochemical (IHC) staining was performed on 3-µm sections obtained from formalin-fixed, paraffin-embedded blocks. Sections were autoclave-pretreated in Histofine antigen retrieval solution (pH 9) (Nichirei, Tokyo, Japan). Endogenous peroxidase activity was blocked using 3% hydrogen peroxide, and the sections were incubated with diluted antibodies over night at 4°C. A subsequent reaction was performed with a biotin-free horseradish peroxidase enzyme-labeled polymer of the Envision Plus detection system (Dako Co, Produktionsvej, Denmark). A positive reaction was visualized with a diaminobenzidine (DAB) solution, followed by counterstaining with Mayer’s hematoxylin. The following primary antibodies were used for the analyses: mouse monoclonal antibody (mAb) against EZH2 (1∶50 dilution; BD Transduction Laboratories, California, USA), mouse mAb against Ki-67 (1∶100 dilution; Dako Co), mouse mAb against p27^Kip1^ (1∶150 dilution; BD Biosciences), rabbit mAb against p21^WAF1^ (1∶50 dilution; Santa Cruz, Dallas, Texas, USA.), rabbit mAb against PTEN (1∶100 dilution; Cell Signaling, Danvers, Massachusetts, USA), mouse mAb against BMI-1(1∶100 dilution; Millipore, Billerica, Massachusetts, USA). Negative controls for immunostaining were prepared by omitting the primary antibody. All of the IHC staining results were independently scored by two pathologists (K.I and Y.H). Five random high-power fields per lesion were evaluated to determine the mean number of EZH2-, BMI-1-, Ki67-, p27^Kip1^-, and p21^WAF1^-positive nuclei and the mean number of PTEN-positive nuclei or cytoplasmic cell number in IPMNs.

### RNA extraction from formalin-fixed paraffin-embedded specimens and real time PCR analyses

Total RNA was successfully extracted from elective 20 IPMN lesions (borderline IPMN in 9, CIS in 6, invasive carcinoma in 5) and 7 pancreatic normal ducts. Formalin-fixed paraffin-embedded block with IPMN lesion or normal pancreatic duct was sectioned into 10-µm, 6–12 serial pieces. IPMN lesions and normal pancreatic ducts were macro-dissectioned in comparison with H&E staining (**Figure S1**). Then, total RNA was extracted using RNeasy FFPE Kit (QIAGEN) according to each instruction manual. Total RNA was converted to cDNA by reverse transcription polymerase chain reaction (RT-PCR). To measure mRNA expression levels of *EZH2* and *p27^Kip1^*, real time PCR assay was performed using LightCycler 480 (Roche) system. Probes and primers were designed using Roche probe library system. GAPDH (glyceraldehyde-3-phosphate dehydrogenase) mRNA was used as reference. The probes and primers were summarized in **Table S1**.

### Statistical analyses

Continuous variables were compared using Student’s *t*-test. Pearson’s correlation coefficient was used to analyze the correlation between the EZH2 p27^Kip1^ or Ki-67 protein expressions. The correlation between *EZH2* mRNA and p27^Kip1^ mRNA was also analyzed using Pearson’s correlation coefficient. Overall survival and disease-free survival were calculated using the Kaplan-Meier method and compared using the log-rank test. Statistical analyses were performed as indicated with Excel Statistics (Social Survey Research Information Co.). Differences were considered significant if *p*<0.05.

## Results

### Pancreatic IPMNs have multifocality and malignant potential with accelerated cell proliferation

**Figure 1:**
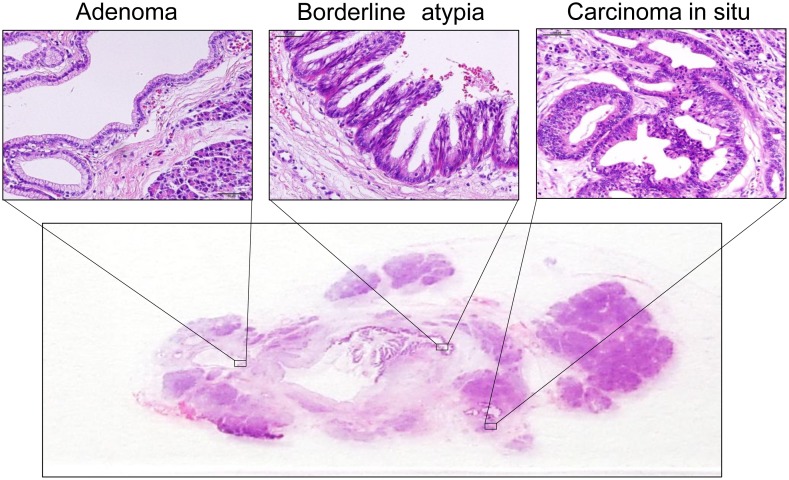
H&E staining of IPMNs. IPMNs consist of different degrees of dysplasia characterized by multifocality.

**Figure 2:**
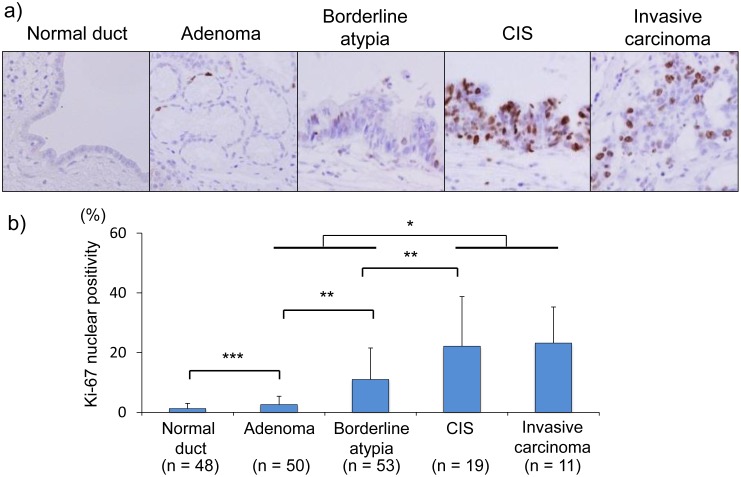
Increased cell proliferative activity during IPMN progression. a) Immunohistochemical staining of Ki-67 in IPMN lesions (×40). CIS, carcinoma in situ. b) The percentage of Ki-67-positive nuclear cells in IPMN lesions. The cell proliferative activity increased during IPMN progression, and the number of Ki-67-positive cells among malignant IPMNs (carcinoma in situ and invasive carcinoma) was significantly higher than that in non-malignant IPMNs (adenoma and borderline atypia) (*p*<0.0001). *, *p*<0.0001; **, *p*<0.001; ***, *p*<0.01.

Our series of 54 resected specimens included a total of 181 lesions (48 normal duct and 133 IPMN lesions), including 50 with adenoma, 53 with borderline atypia, 19 with CIS, and 11 with invasive carcinoma. Thus, pancreatic IPMNs were characterized by multifocal and malignant potential represented by an adenoma-carcinoma sequence (**Figure 1**). Next, we assessed proliferative activity, which is thought to be a crucial factor in an adenoma-carcinoma sequence, in IPMNs by Ki-67 immunostaining. The mean counts of Ki-67-positive nuclei in normal duct and during IPMN progression (adenoma, borderline atypia, CIS, and invasive carcinoma) were 1.29%, 2.63%, 11.04%, 22.18%, and 23.23% respectively. Whereas normal pancreatic ductal cells contained few Ki67-positeve nuclei, in the number of Ki67-positive nuclei in IPMNs increased in proportion to the aggressiveness of the IPMNs (**Figure 2a**). Indeed, the cell proliferative activity in malignant IPMNs (CIS and invasive carcinoma) was significantly higher than that in non-malignant IPMNs (adenoma and borderline atypia) (*p*<0.0001) (**Figure 2b**). Thus, the cell proliferative activity in pancreatic IPMNs was increased during the disease progression of IPMNs, particularly in the shift from nonmalignant- to malignant-IPMNs suggesting that accelerated tumor-proliferative activity plays an important role in the adenoma-carcinoma sequence of pancreatic IPMNs.

### Tumor suppressor p27^Kip1^ is downregulated according to the IPMN progression

**Figure 3:**
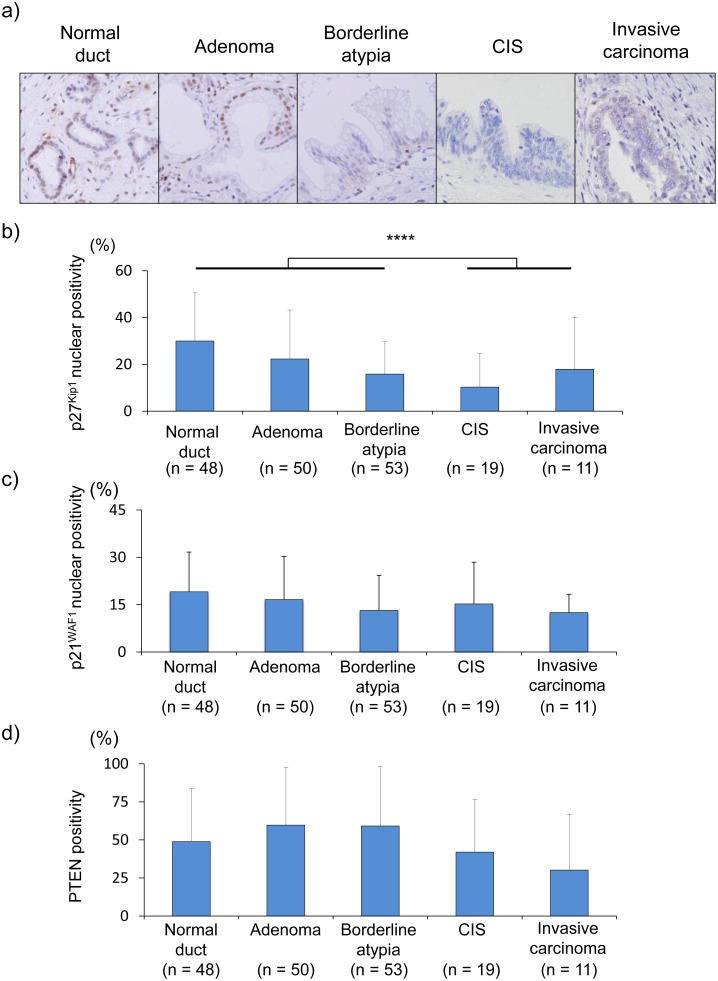
The expression patterns of p27^Kip1^, p21^WAF1^, and PTEN in normal duct and IPMN lesions. a) Immunohistochemical staining of p27^Kip1^ in IPMN lesions (×40). CIS, carcinoma in situ. b) The percentage of p27^Kip1^-positive nuclear cells in IPMN lesions. ****, *p*<0.05. c) The percentage of p21^WAF1^-positive nuclear cells in IPMN lesions. d) The percentage of PTEN-positive cells in IPMN lesions.

EZH2 has been reported to promote cell proliferation via downregulation of tumor suppressor protein such as p27^Kip1^ [18], [23] and p21^WAF1^ [24]. PTEN has been also reported to be a tumor suppressor protein though its association with EZH2 is unknown. To assess the mechanism of the accelerated cell proliferative activity during the pancreatic IPMN progressions, the expressions of *p27^Kip1^*, *p21^WAF1^*, and *PTEN* tumor suppressor proteins was examined by immunohistochemical staining (**Figure 3**). Whereas p27^Kip1^ exhibited strong nuclear localization in the normal pancreatic ductal cells, p27^Kip1^ in IPMN lesions was down-regulated in proportion to IPMN progression (**Figures 3a and b**). The mean counts of p27^Kip1^-positive nuclei in normal duct, adenoma, and borderline atypia were 30.0%, 22.3%, and 15.9%, respectively, whereas those in CIS and invasive carcinoma were 10.3% and 18.0% respectively. The p27^Kip1^-nuclear positive ratio in CIS was the lowest during IPMN progression, and the p27^Kip1^-nuclear positive ratio (13.1%) in malignant lesions (CIS and invasive carcinoma) was significantly lower than that (22.4%) in normal and non-malignant lesions (normal duct, adenoma, and borderline atypia) (*p = *0.0158). Thus, p27^Kip1^ protein expression in IPMN lesions was significantly downregulated with the progression of IPMN, suggesting the downregulation of tumor suppressor protein p27^Kip1^ plays a crucial role in pancreatic IPMN progression. Next, p21^WAF1^ was examined. The p21^WAF1^ nuclear-positive ratio (19.1**%**) in normal pancreatic ductal cells was the highest, and then, the ratio gradually decreased, reaching a nadir in invasive carcinoma during IPMN progression (**Figure 3c**). However, there was no significant difference in p21^WAF1^ protein expression between non-malignant and malignant IPMNs. Phosphatase and tensin homolog deleted from chromosome 10 (PTEN) is a lipid phosphatase that antagonizes the action of phosphatidylinositol-3 kinase (PI3K), and thereby acts as tumor suppressor gene, and the relation between EZH2 and PTEN has not been unclear now. PTEN protein was expressed in both of the nucleus and cytoplasm, and it was broadly detectable from normal pancreatic ductal cells to invasive carcinoma by IHC. Whereas PTEN protein in non-malignant IPMNs slightly increased compared with the normal duct, in malignant IPMNs, it decreased to less than that in the normal pancreatic duct. PTEN exhibited a different expression pattern than Ki-67-positive nuclei (**Figure 3d**). Collectively, among p27^Kip1^, p21^WAF1^, and PTEN, the down-regulation of p27^Kip1^ appeared to be the crucial factor in the accelerated proliferative activity during IPMN progression.

### Overexpression of EZH2 may be associated with downregulated p27^Kip1^ expression and accelerated cell proliferative activity in malignant IPMNs

**Figure 4:**
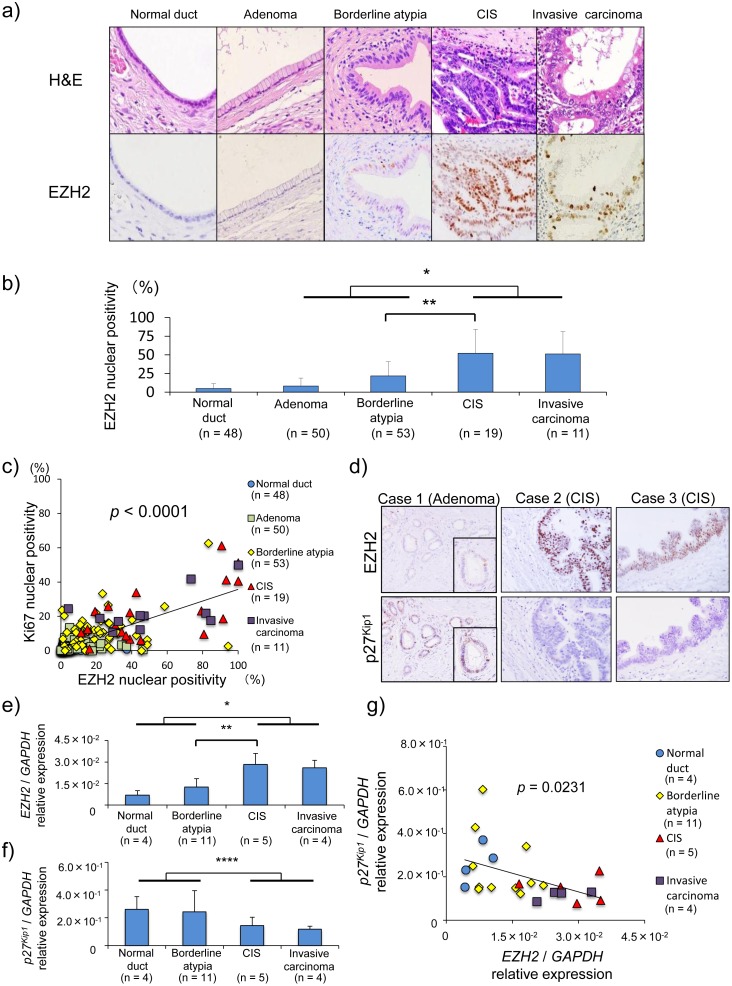
Increased EZH2 expression during IPMN progression is associated with p27^Kip1^ downregulation and high proliferative activity. a) H&E and immunohistochemical staining of EZH2 in the corresponding IPMN lesions (×40). CIS, carcinoma in situ. b) The percentage of EZH2 positive nuclear cells in IPMN lesions. EZH2 expression was upregulated according to the progression of IPMNs, and the number of EZH2-positive cells in malignant IPMN (carcinoma in situ and invasive carcinoma) was significantly higher than that in non-malignant IPMN (adenoma and borderline atypia). c) The positive association between the percentage of EZH2 nuclear-positive cells and the percentage of Ki-67 nuclear-positive cells (*p*<0.0001, R^2^ = 0.5226). d) Inverse expression patterns of EZH2 and p27^Kip1^ in non-malignant (case 1 with adenoma) and malignant (cases 2 and 3 with carcinoma in situ) IPMN lesions using serial sections (×40). e) EZH2 mRNA expression in IPMN lesions and normal ducts. f) p27^Kip1^ mRNA expression in IPMN lesions and normal ducts. g) Inverse association between *EZH2* and *p27^Kip1^* mRNA levels (*p = *0.0231, R^2^ = 0.2131). *, *p*<0.0001; ***, *p*<0.01; ****, *p*<0.05.

To further assess the underlying molecular mechanism of the downregulation of p27^Kip1^ during pancreatic IPMN progression, we focused on the alteration of EZH2 and BMI-1, which function as transcriptional repressors during tumorigenesis. Whereas EZH2 nuclear expression was negative in the normal pancreatic ductal cells, EZH2 expression in IPMN lesions increased during IPMN progression, particularly from borderline atypia to invasive carcinoma (**Figures 4a and b**). The EZH2 nuclear-positive ratio in malignant IPMNs was significantly higher than that in non-malignant IPMNs (*p*<0.0001).

Furthermore, EZH2 expression level in IPMN lesions was positively correlated with the Ki-67 nuclear-positive ratio (*p*<0.0001, R^2^ = 0.5226) (**Figure 4c**). This result shows increased EZH2 expression was associated with the high proliferative ability in malignant IPMNs.

Immunohistochemical staining for EZH2 and p27^KIP1^ was performed on serial sections to determine whether the increased EZH2 expression, which acts as an epigenetic gene silencer, was associated with the downregulated p27^Kip1^ expression during IPMN progression (**Figure 4d**). p27^Kip1^-positive cells were EZH2-negative in non-malignant IPMN (adenoma). In contrast, EZH2-positive cells in malignant IPMN (CIS lesions) displayed negative expression of p27^Kip1^ protein.

To ascertain the relationship between EZH2 and p27^Kip1^, mRNA levels were examined in elective 20 IPMN (borderline IPMN in 11, CIS in 5, invasive carcinoma in 4) lesions and 4 pancreatic normal ducts. *EZH2* mRNA expressions in malignant lesions were significantly approximately 2.5 times higher than those in benign lesions (*p*<0.0001) (**Figure 4e**). In contrast, *p27^Kip1^* mRNA in malignant lesions was significantly decreased approximately by half compared to those of benign lesion (*p*<0.05) (**Figure 4f**), and there was an inverse correlation between *EZH2* and *p27^Kip1^* mRNA levels (R^2^ = 0.213, *p* = 0.0231) (**Figure 4g**). By immunohistochemical staining, EZH2 nuclear positivity revealed the similar negative tendency with p27^Kip1^ nuclear positivity, whereas it did not reach the significant difference. (**Figure S2**).

**Figure 5:**
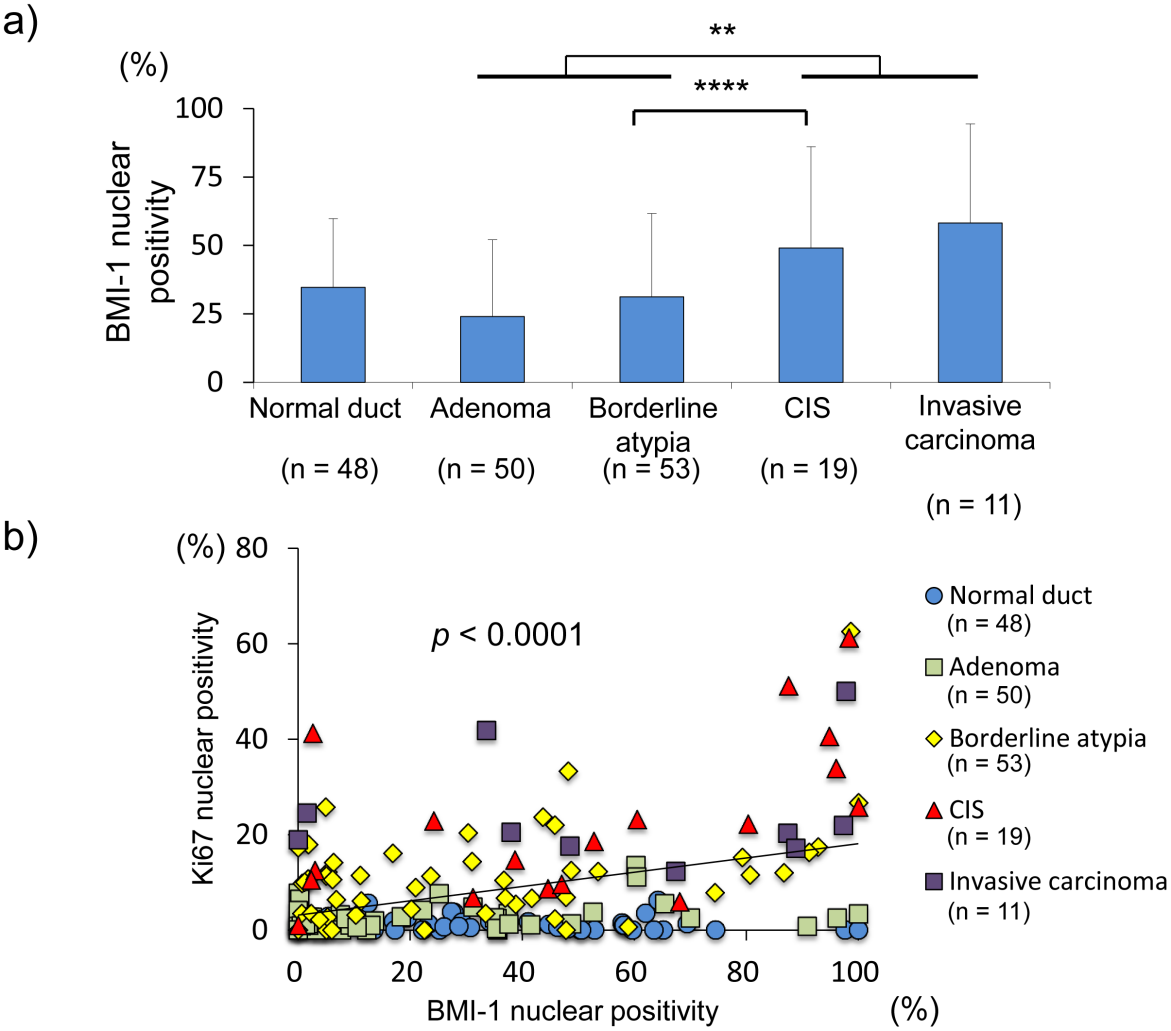
Percentage of BMI-1 nuclear-positive cells in IPMN lesions. a) The number of BMI-1-positive cells in malignant IPMN (carcinoma in situ and invasive carcinoma) was significantly higher than that in non-malignant IPMN (adenoma and borderline atypia) (*p* = 0.042). CIS, carcinoma in situ. **, *p*<0.001; ****, *p*<0.05. b) The positive association between the percentage of BMI-1 nuclear-positive cells and the percentage of Ki-67 nuclear-positive cells (*p*<0.0001, R^2^ = 0.1586).

Collectively, these data suggest that increased EZH2 expression during pancreatic IPMN progression may result in the transcriptional silencing of p27^Kip1^ and may thereby be associated with accelerated proliferative activity. Additionally, we examined the expression of BMI-1 (B lymphoma Moloney murine leukemia virus insertion region 1 homolog), another polycomb protein, during pancreatic IPMN progression, since BMI-1 plays an important role in cancer cell proliferation via transcriptional repression [25], [26]. BMI-1 protein expression was detectable in pancreatic normal duct and then gradually and moderately increased during IPMN progression. Although the BMI-1 expression in malignant IPMNs was significantly higher than that in non-malignant IPMNs (**Figure 5a**), similar to EZH2 expression, the correlation between BMI-1 and Ki-67 expression in IPMN lesions (**Figure 5b, ***p*<0.0001, R^2^ = 0.1587) revealed a weaker positive correlation compared to that between in EZH2 and Ki-67 expression (*p*<0.0001, R^2^ = 0.5226). These findings suggest that EZH2 rather than BMI-1 aberration is deeply associated with the accelerated proliferative activity during IPMN progression.

## Discussion

**Figure 6:**
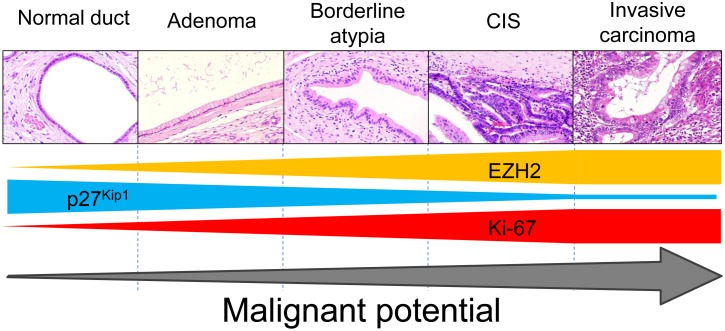
Schema of the epigenetic alterations during pancreatic IPMN progression. Whereas EZH2 is overexpressed during pancreatic IPMN progression, p27^Kip1^ is inversely downregulated, which leads to accelerated cell proliferation in the malignant IPMN lesions. CIS, carcinoma in situ.

Here, we demonstrate that EZH2 alteration promotes the acquisition of malignant potential during pancreatic IPMN progression. Inversely, tumor suppressor p27^Kip1^ was undetectable in EZH2-positive cells, and its expression was silenced during IPMN progression. Thus, EZH2 expression in pancreatic IPMNs was associated with the high proliferative ability in malignant IPMNs due to its downregulation of p27^Kip1^. Collectively, EZH2 plays an important role in acquiring malignant potential via p27^Kip1^ silencing (**Figure 6**).

Oncogenesis is a multistep process associated with genetic and epigenetic alterations. Genetic alterations in the KRAS and TP53 genes are frequently observed in pancreatic IPMNs, and arise gradually during IPMN progression. KRAS mutations are detectable even in IPMNs without dysplasia and increase with the grade of dysplasia, suggesting that KRAS mutation is an early event in IPMN lesions [11], [27]. In contrast *TP53* mutations in IPMNs are undetectable in IPMN hyperplasia or adenoma, whereas they become detectable in IPMN carcinoma at a rate of 38% to 50% [28], [29]. Thus, *TP53* mutation is typically observed in malignant IPMNs such as CIS and invasive carcinoma [13], and these mutations are considered a late event in IPMN lesions. In the present study, the EZH2 expression level in adenoma was similar to that in normal pancreatic duct, and it increased during IPMN progression. EZH2 was significantly upregulated during the malignant step from borderline atypia to CIS, suggesting that EZH2 overexpression is a late event in pancreatic IPMNs and plays an important role in the malignant step of IPMNs. The oncogenesis of pancreatic IPMNs may be a complex process associated with the accumulation of genetic and epigenetic alterations.

In the present study, among tumor suppressor proteins *p27^Kip1^*, *p21^WAF1^*, and *PTEN*, p27^Kip1^ was found to be an EZH2-related negative regulator of cell proliferative activity in pancreatic IPMNs. Furthermore, the p27^Kip1^ expression level was the lowest in in malignant IPMN (CIS and invasive carcinoma) compared to the other stages. Indeed, EZH2 expression and cell proliferative activity had an inverse pattern from p27^Kip1^ expression and displayed the highest expression in CIS. These findings suggest that the EZH2-mediated accelerated cell proliferation via p27^Kip1^ downregulation played a crucial role in the malignant step from borderline atypia to CIS rather than in the progression from CIS to invasive carcinoma. In contrast, PTEN expression exhibited a different expression pattern from EZH2 and cell proliferative activity. Whereas PTEN was slightly increased in non-malignant IPMNs compared with normal pancreatic duct, in malignant IPMNs, PTEN decreased to a lower level than in the normal pancreatic duct. These findings suggest that the disruption of upregulated PTEN expression in non-malignant IPMN may lead to progression into malignant IPMN. The role of PTEN in IPMN progression remains unknown and should be clarified in future research.

This study is the first to find that epigenetic alteration is associated with malignant behavior during pancreatic IPMN progression, suggesting that a strategy to reverse the epigenetic gene silencing process could prevent IPMN progression. An important, limitation of this study, is that we found no significant difference in long-term surgical outcomes between patients with high and low expression of EZH2 (*p* = 0.1408), because there were only three patients whose cause of death was pancreatic IPMN after surgery. Therefore, the clinical impact of EZH2 expression as a prognostic factor remains unclear. Further studies including a large population of patients with pancreatic IPMN are needed to clarify the clinical impact of EZH2 as a prognostic factor.

In conclusion, EZH2-mediated p27^Kip1^ downregulation is associated with the accelerated cell proliferation and malignant step in pancreatic IPMN.

## Supporting Information

Figure S1 **Macro-dissection of representative IPMN lesion.** IPMN lesions and pancreatic normal duct on 10-µm section was dissected using sterilized needle and razor. Hematoxylin-eosin staining was performed before dissected and after dissected section to clarify the procedure of dissection.
(TIF)

Figure S2 **The inverse relation between EZH2 nuclear positivity and p27 nuclear positivity.**
(TIF)

Table S1 **Probe and primer for real time PCR analysis.**
(DOCX)
