# Supplementary figures and images for: EZH2 Is Associated with Malignant Behavior in Pancreatic IPMN via p27Kip1 Downregulation
Source: PLoS One. 2014 Aug 1;9(8):e100904. doi: 10.1371/journal.pone.0100904 (PMC4118850; doi:10.1371/journal.pone.0100904)

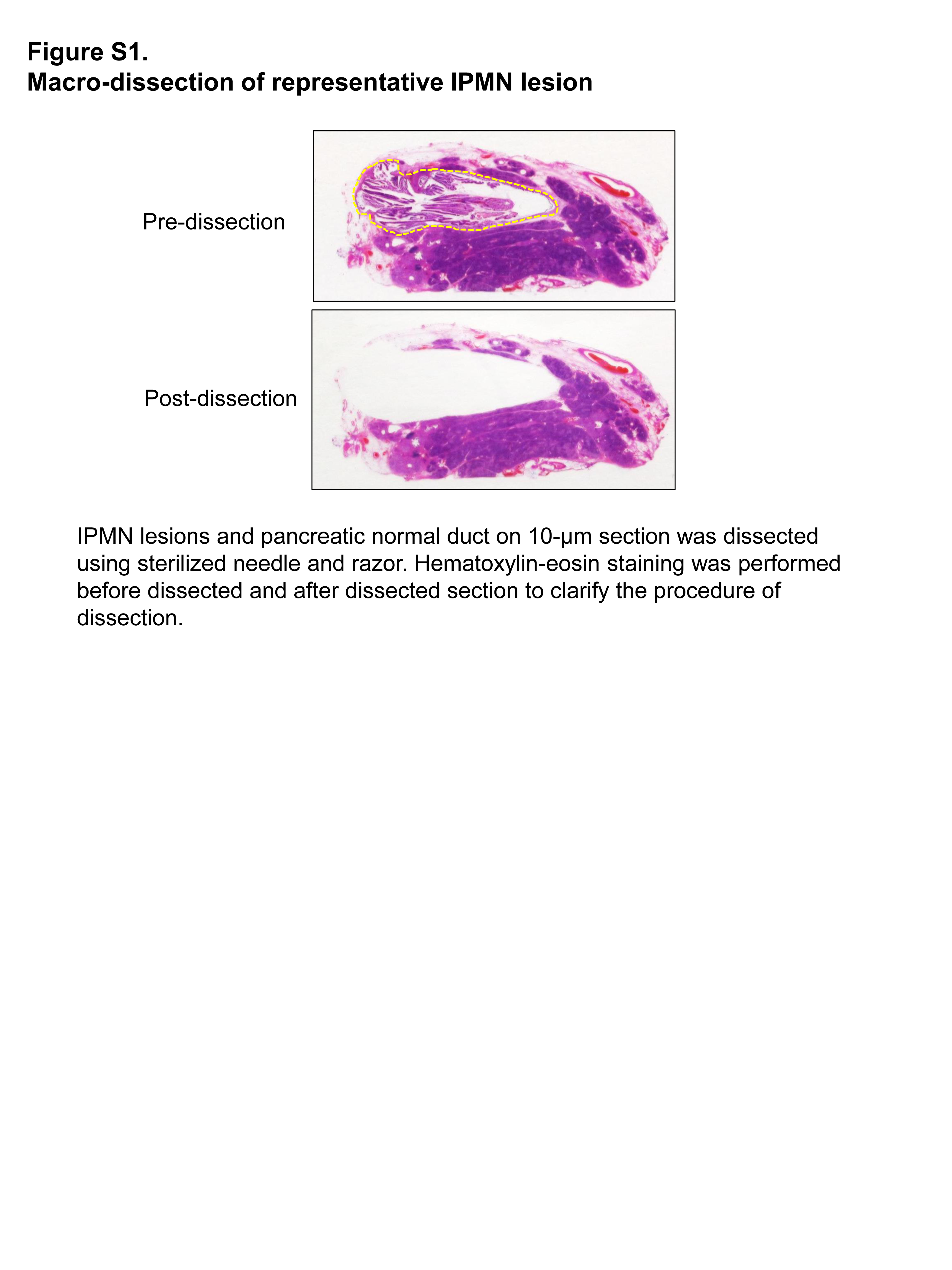

Supplement: Figure S1 — Macro-dissection of representative IPMN lesion. IPMN lesions and pancreatic normal duct on 10-µm section was dissected using sterilized needle and razor. Hematoxylin-eosin staining was performed before dissected and after dissected section to clarify the procedure of dissection. (TIF) [file pone.0100904.s001.tif]

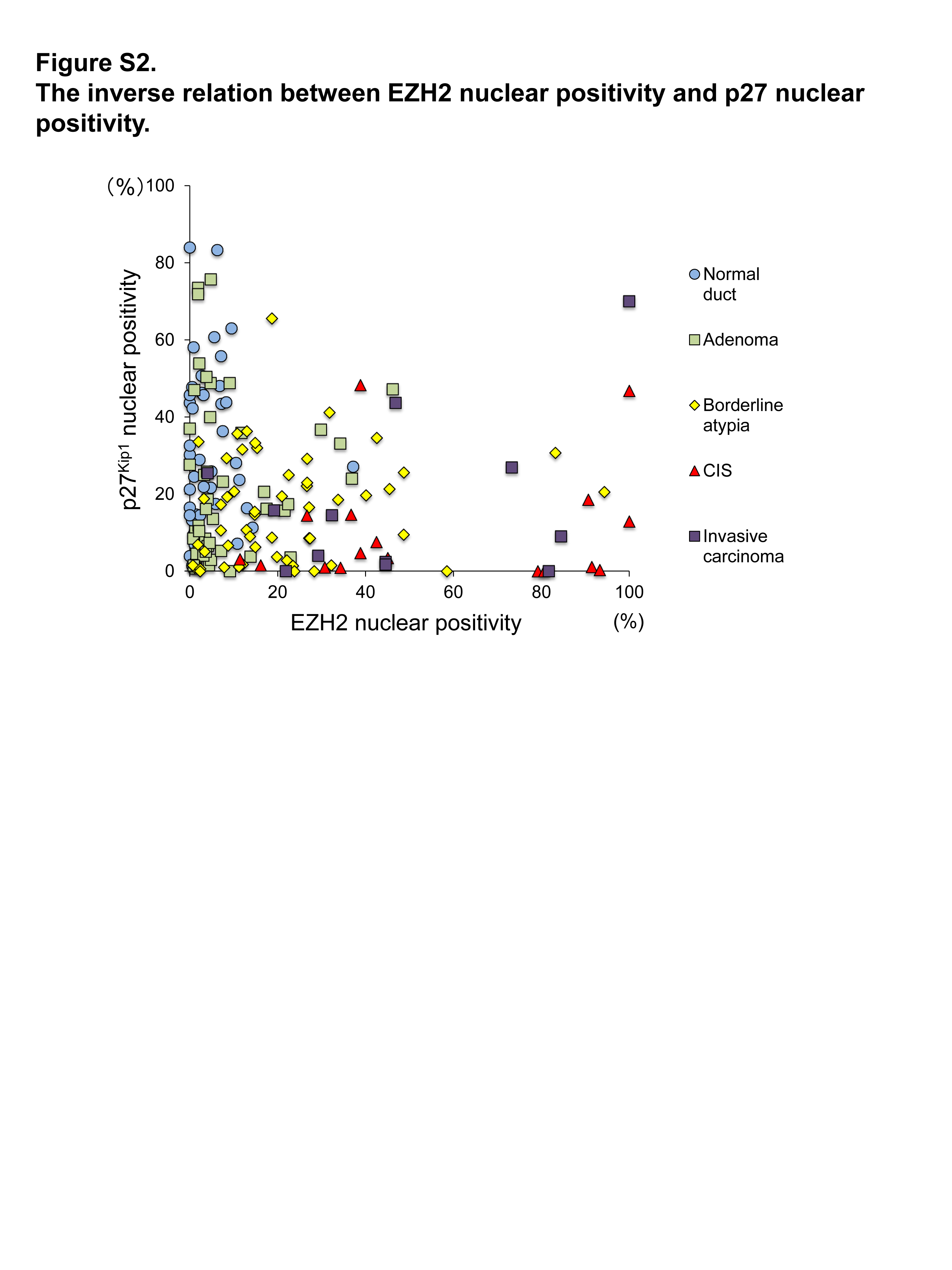

Supplement: Figure S2 — The inverse relation between EZH2 nuclear positivity and p27 nuclear positivity. (TIF) [file pone.0100904.s002.tif]
