# Supplementary material for: EZH2 Is Associated with Malignant Behavior in Pancreatic IPMN via p27Kip1 Downregulation
Source: PLoS One. 2014 Aug 1;9(8):e100904. doi: 10.1371/journal.pone.0100904 (PMC4118850; doi:10.1371/journal.pone.0100904)
Supplement: Table S1 — Probe and primer for real time PCR analysis. (DOCX) [file pone.0100904.s003.docx]

**Table S1.**

**Probe and primer for real time PCR analysis**

| **Gene name** | **Probe number** | **Primer (forward)** | **Primer (reverse)** |
| --- | --- | --- | --- |
| *EZH2* | #64 | gactggcgaagagctgtttt | tctttcgatgccgacatactt |
| *p27^Kip1^* | #39 | ccctagagggcaagtacgagt | agtagaactcgggcaagctg |
| *GAPDH* | #60 | agccacatcgctcagacac | gcccaatacgaccaaatcc |
